# Prediction of optical properties of rare-earth doped phosphate glasses using gene expression programming

**DOI:** 10.1038/s41598-024-66083-0

**Published:** 2024-07-05

**Authors:** Fahimeh Ahmadi, Raouf El-Mallawany, Stefanos Papanikolaou, Panagiotis G. Asteris

**Affiliations:** 1 Institute of Research and Development, Duy Tan University, Da Nang, 550000 Vietnam; 2 Physics Department, Faculty of Science, Menoufia University, Shebin El Kom, Egypt; 3 NOMATEN Centre of Excellence, National Center for Nuclear Research, ul. A. Soltana 7, 05-400 Swierk/Otwock, Poland; 4 Department of Nuclear Science and Engineering, Massachusetts Institute of Technology, 77 Massachusetts Avenue, Cambridge, MA 02139 USA; 5 Computational Mechanics Laboratory, School of Pedagogical and Technological Education, Athens, Greece

**Keywords:** Phosphate glass, Rare-earth ions, Optical properties, Judd–Ofelt parameters, Gene expression programming, Materials science, Optics and photonics

## Abstract

The progression of optical materials and their associated applications necessitates a profound comprehension of their optical characteristics, with the Judd–Ofelt (JO) theory commonly employed for this purpose. However, the computation of JO parameters (Ω_2_, Ω_4_, Ω_6_) entails wide experimental and theoretical endeavors, rendering traditional calculations often impractical. To address these challenges, the correlations between JO parameters and the bulk matrix composition within a series of Rare-Earth ions doped sulfophosphate glass systems were explored in this research. In this regard, a novel soft computing technique named genetic expression programming (GEP) was employed to derive formulations for JO parameters and bulk matrix composition. The predictor variables integrated into the formulations consist of JO parameters. This investigation demonstrates the potential of GEP as a practical tool for defining functions and classifying important factors to predict JO parameters. Thus, precise characterization of such materials becomes crucial with minimal or no reliance on experimental work.

## Introduction

In recent times, there has been extensive exploration of the optical absorption and luminescence characteristics of rare earth ions (REIs) doped glasses based on borate, silicate, phosphate, and tellurite compositions. There is high demand for these materials in many technological and commercial applications, containing fluorescent display technology, optical detectors, bulk lasers, optical fibers, waveguide lasers, and optical amplifiers^1–4^. Notably, REIs such as Eu^3+^, Sm^3+^, Dy^3+^, Er^3+^, and Pr^3+^ are commonly utilized for the development of various optical devices^5,6^. To obtain efficient luminescence from REIs, a suitable glass host must be carefully selected.

A significant amount of attention has been given to phosphate glasses compared to silicate and borate glasses. This preference is attributed to their distinctive features, including high transparency, low melting point, high thermal stability, and high gain density^7,8^. These characteristics primarily stem from the notable solubility of RE ions, along with low refractive index and dispersion. Further, sulfate ions dissolve readily in the phosphate glass matrix.

These glass systems form dithiophosphate (DPT) molecules due to the relatively poor interaction between sulfate and metaphosphate ions. Since sulfate and phosphate ions interact weekly and inconsistently, many REIs can be incorporated into this process. Consequently, it is anticipated that this glass system will enable high efficiency of luminescence with low non-radiative losses.

The Judd–Ofelt (JO) theory has emerged as a highly consequential framework with extensive applications in chemistry, material science, and related academic disciplines. These applications encompass solid-state lasers^9,10^, thermal sensors^11,12^, optical amplifiers, up-conversion^13^, and diverse biological contexts^14,15^. The JO theory is really handy because it helps us understand how materials interact with light, like how likely they are to absorb or emit light, and how they behave when they do. But it's not something you can just pick up easily; you need to know a lot about how solid materials and quantum stuff work. Plus, getting the data needed for JO theory involves making very specific materials and doing lots of precise experiments, which takes a long time. And on top of all that, you also have to measure how the material absorbs light to get the right info for JO theory and other tests.

The JO theory is elegant but often tough to work with because of all the steps involved in making materials, measuring them, crunching the numbers, and analyzing the results. Despite these challenges, there's a good reason to look for simpler ways to get the same kind of info, especially since JO theory has so many useful applications. That's why we've come up with a new way to figure out JO parameters indirectly, using a method called GEP, in glasses doped with RE^3+^ ions. This could make it a lot easier for scientists to get the data they need, potentially changing the way we do research in this field.

Because artificial intelligence (AI) approaches can create nonlinear correlations between input and output data, they have become increasingly popular in numerous science and engineering disciplines^16–22^. A significant body of research has been dedicated to leveraging AI approaches for predicting structural properties of glasses^23–27^. Gaafar et al.^25^ employed the artificial neural network (ANN) method to forecast critical parameters for roughly thirty glass compositions, such as moduli of elasticity, density, and ultrasonic-wave velocities. The anticipated outcomes showed agreement with experimentally determined parameters. Additionally, they used an AI model to simulate ultrasonic wave velocities, density, and elastic moduli for different tellurite glasses, obtaining consistency between experimental and predicted results^28^. Their model was further utilized in the manufacturing of four niobium-lead-tellurite glass systems, where experimental findings indicated that Nb_2_O_5_ serves in the role of a framework modifier, contributing oxygen ions to form [TeO_3_] trigonal pyramids from [TeO_4_] trigonal bi-pyramids. Furthermore, using a large dataset, Deng carried out a thorough machine-learning analysis to estimate the density of oxide glasses as well as Young's modulus, shear modulus, and Poisson's ratio^29^.

This paper focuses on the investigation of twelve different series of sulfophosphate glass, comprising 51 glass samples synthesized through the melt-quenching method. Additionally, 20 glass samples were collected from the literature. The study introduces a GEP model designed to predict JO parameters for the total of 70 samples. This GEP model enables indirect measure of JO parameters, eliminating the need for expensive oxide materials.

## Research significance

The estimation of the optimal optical properties for rare-earth doped phosphate glasses is a challenge engineering task due to its multidimensional nature, involving twelve input and three output parameters. Classical computational techniques such as regression analysis are insufficient for addressing this complexity, leaving a gap in our understanding of these materials' behavior. This complexity arises from the diverse range of parameters influencing the optical characteristics, spanning from the bulk matrix composition to the presence of rare-earth dopants.

Given this complexity, surrogate soft computing methods such as Gene Expression Programming (GEP) emerge as essential tools for tackling such challenges. Unlike classical techniques, GEP excels in capturing the nonlinear relationships inherent in these multidimensional problems. This study utilizes GEP to reveal the complex and strongly nonlinear nature of predicting optical properties, with co-authors from both materials science and machine learning fields contributing their knowledge.

The significance of this research has mainly to do with its potential to contribute to more reliable estimation of optical properties, reducing the need for extensive experimental work. By developing GEP-based surrogate mathematical models, this study aims to unveil the fundamental relationships between input and output parameters, contributing to a holistic design and development of novel optical materials. Moreover, by emphasizing the importance of reliable and sufficient databases, this research underscores the crucial role of data quality in computational modeling, further emphasizing the multinstitutional and interdisciplinary collaboration such as experts from materials science and data science learning. Summarizing, this study not only addresses the challenge in materials science but also demonstrates the great importance of metaheuristic computational techniques such as GEP which provide us analytical formulas. By bridging the gap between theory and experiment, the derived and proposed closed form GEP-based equations paves the way for accelerated innovation in the field of optical materials, showcasing the collaborative efforts of researchers from diverse scientific backgrounds and supporting corresponding academic lectures on the subject.

## Gene expression programming

Gene Expression Programming (GEP), devised by Ferreira^29^, represents a revolutionary method for developing mathematical models. Based on the principles of evolutionary computation inspired by inherent evolution, GEP provides a solution in the shape of a tree configuration generated from a specific dataset. The fundamental genetic material in GEP is characterized by linear chromosomes comprised of genes which architecturally structured into a head and a tail. These chromosomes serve as a genome and undergo modifications through processes such as mutation, root and gene transposition, gene recombination, and also one and two-point recombination^30^.

The unique feature of GEP lies in the encoding of expression trees within these chromosomes, which become the subject of selection. This separation into distinct entities, the genome, and the expression tree, with specialized functions, contributes to the algorithm's exceptional efficiency, surpassing existing adaptive techniques. The GEP algorithm unifies the predominant aspects of two preceding inheritance algorithms, namely genetic algorithms and genetic programming, with the aim of overcoming their respective limitations. In GEP, the chromosome genotype mirrors that of a genetic algorithm, while the phenotype takes the form of a tree structure that varies in size and length, akin to genetic programming. By overcoming the constraints of earlier algorithms regarding the double function of chromosomes, GEP ensures the sustained health of offspring chromosomes through multiple genetic operators, achieving faster rates than genetic programming^31^.

The logical interdependence among multiple variables, if present, may be encapsulated within a function, potentially an accurately describable one. This function can encompass algebraic operators such as + , −, *, /, Boolean logic operators including OR, AND, and IF, or a diverse range of algebraic functions. Clearly, the scrutiny of the logical connection among variables is imperative^32^. In the application of GEP algorithm to discern a relationship between variables a and b with y, a linear chromosomes population is initially generated. In these chromosomes, each position of the genes can accommodate one of the variables. Once the chromosomes are constructed and populated with variables, the subsequent step involves evaluating the fitness of each individual (chromosome) within the given generation in which chromosomes are expressed as expression trees (ET). Analogous to a protein in a natural cell that dictates a gene's phenotype, an ET serves as a representation of the chromosome's structure and function. This process facilitates the exploration and understanding of the logical relationships among variables by embodying them in a tree-like structure, aiding in the comprehensive analysis of complex functions and their dependencies.

Ferreira^29^ introduced an ingenious and effective language known as Karva for the expression of genes and the generation of ETs. In this system, a mathematical equation or program is formulated and obtained from each chromosome. These chromosomes are comprised of random terminals and functions, providing a structured representation of genetic information.

To evaluate the performance of these chromosomes, fitness is determined by comparing the calculated value of y through the equation against the actual values for specified points of a and b, given in fitness cases. The closeness of the calculated y values to the actual values at different points signifies the accuracy of the equation, and a smaller difference results in higher fitness.

In the initial generation, fitness is computed for each chromosome, and their scores contribute to the selection process for the next generation, proportional to their overall fitness. Additionally, the fittest individual in any generation, without undergoing the procedure of selecting, is directly carried over to the next generation. This methodology ensures the continual refinement of the population, emphasizing the preservation of superior genetic material for subsequent generations in the evolutionary process.

In the progression to the subsequent generation, genotype as the linear state of the chromosomes from the current generation is employed. This entails the presence of full-length chromosomes, irrespective of whether they are active or inactive, in the subsequent generation. Notably, the inactive part of a gene in the current generation may undergo activation, becoming a fully adaptable component through a mutation in the next generation.

Defining the functions, terminals, fitness function, linking function, chromosomes’ structure as well as determining the features of the operators and ultimately implementing the algorithm are the fundamental steps in designing a GEP algorithm. The initial step in generating the subsequent generation involves the replication process, which is facilitated by the Roulette Wheel method. Conceptually, the wheel rotates and selects a chromosome at each turn, a process executed by creating and allocating random numbers. Higher rated chromosomes, determined by their fitness, have a greater likelihood of being chosen. Importantly, the selection process is akin to the random selection observed in natural evolution, bringing the algorithm closer to this fundamental aspect.

This replication procedure continues until the specified number of chromosomes from the current generation is transferred to the next, maintaining a consistent number of chromosomes throughout the evolutionary process. This perpetuates the genetic diversity and adaptability of the population over successive generations. Following the replication process, the restructuring phase commences, signifying the sequential application of genetic operators on identical chromosomes in the prescribed order outlined in the algorithm. This sequential transformation of chromosomes mirrors the natural evolution process, gradually converging towards an ideal equation of interest after a series of generations.

**Figure 1:**
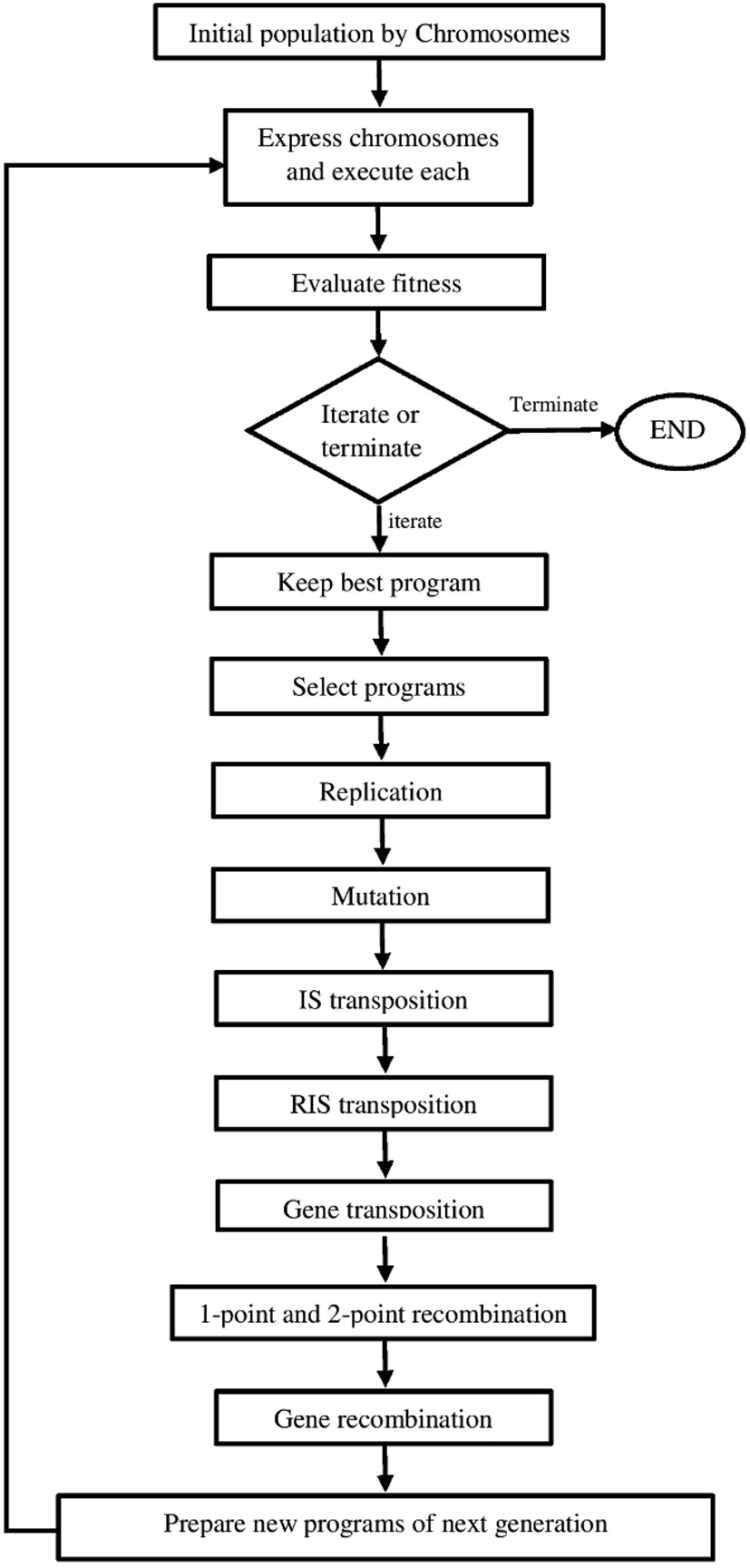
The GEP flowchart.

In this iterative process, new-generation chromosomes are generated, and their successive assessment ensures the continual refinement of the population. This simulation of natural evolution through the application of genetic operators enhances the adaptability and performance of the chromosomes over time. To manage computational resources effectively, a limitation can be assigned for the iterations number of the algorithm. This precautionary measure prevents excessive memory and time consumption, allowing for the termination of the algorithm if it fails to recover or converge to a satisfactory solution within a specified timeframe. Figure 1 depicts the flowchart of the GEP algorithm, illustrating the sequential steps involved in the replication, restructuring, and evaluation processes that collectively simulate the dynamics of natural evolution.

## Materials and methods

In this paper, three models were developed to predict the JO parameters of Ω_2_, Ω_4_ and Ω_6_ for phosphate glass compositions using the GEP method. With this models, these important aspects can be calculated while avoiding the utilize of costly oxide materials.

### Judd–Ofelt theory

Determining the absorption band strengths in spectroscopic studies of RE systems is usually a difficult task. The intensities of the absorption bands are determined in terms of oscillator strength by,

1 $${f}_{expt}=\frac{2303m{c}^{2}}{\pi {e}^{2}{N}_{0}}\int \varepsilon (v)dv$$

where m presents the electron mass and e is the electron charge, c is the velocity of light, ${N}_{0}$ denotes to Avogadro’s number, and ε(v) denotes to the molar extinction coefficient which can be calculated by the Beer–Lambert law as,

2 $$\varepsilon \left(v\right)=\frac{{\text{log}}_{10}^{(\frac{{I}_{0}}{I})} }{Cd}$$

where ${\text{log}}_{10}^{(\frac{{I}_{0}}{I})}$ is the absorbance measured at the wavenumber $v$ (cm^−1^), C denotes to the concentration of the lanthanide ions, and d is the length of sample’s light path^33,34^. Based on Judd–Ofelt theory^35,36^, the oscillator strength for the $aJ\to b{J}{\prime}$ transition can be derived by,

3 $${f}_{cal}={P}_{ed}+{P}_{md}=\frac{8{\pi }^{2}mcv}{3h\left(2J+1\right){e}^{2}{n}^{2}}({x}_{ed}{S}_{ed}+{x}_{md}{S}_{md})$$

where ${x}_{ed}$ and ${x}_{md}$ are

4 $${x}_{ed}=\frac{{n\left({n}^{2}+2\right)}^{2}}{9}$$

5 $${x}_{md}={n}^{3}$$

The following equations describe the electric and magnetic dipole line strengths:

6 $${S}_{ed}(aJ,b{J}{\prime})={e}^{2}\sum_{t=\text{2,4},6}{\Omega }_{t}{\left|\langle aJ|{U}^{t}|b{J}{\prime}\rangle \right|}^{2}$$

7 $${S}_{md}(aJ,b{J}{\prime})=\frac{{e}^{2}}{4{m}^{2}{c}^{2}}{\left|\langle aJ|L+2S|b{J}{\prime}\rangle \right|}^{2}$$

It is essential to mention that those magnetic dipole transitions can contribute to the oscillator strength that satisfy the selection rules as $\Delta S=\Delta L=0, \Delta J=0,\pm 1$. By means of a least-square fit to the values of measured oscillator strengths, The Judd–Ofelt intensity parameters can be obtained. It is assumed that the reduced matrix elements of ${\left|\langle aJ|{U}^{t}|b{J}{\prime}\rangle \right|}^{2}$ are constant from host to host^37^. Root mean square error (RMSE) can be used to evaluate the accuracy of the fit,

8 $$RMSE={\left[\frac{\sum {({P}_{calc}-{P}_{exp})}^{2}}{(\varepsilon -3)}\right]}^\frac{1}{2}$$

where $\varepsilon$ denotes to the number of considered transitions in the calculation.

### Experimental setup

#### Principles during the compilation of databases

In this section, a detailed and comprehensive presentation of the experimental procedures conducted to study the optical properties of rare-earth doped phosphate glasses, which will be utilized for constructing the experimental database, is provided. This experimental database will be employed for the training of soft computing models such as Gene Expression Programming-based models, which will be utilized herein.

Before the presentation of both the experimental methods and corresponding results, it is worth emphasizing that the majority of researchers paid significant attention and care to the computational method to be used. However, they often underestimate the importance of the database used for the development, design, and training of forecasting mathematical computational models. The authors of this study strongly believe that the reliability of a predictive model, which should be the flagship concern, depends primarily on the reliability and adequacy of the database, without ignoring the importance of the computational method and technique to be applied.

Moreover, to avoid any misinterpretation, the term "reliable and adequate" refers to a database in which the data are both reliable (true) and statistically sufficient. Statistically sufficient means that the database covers smooth distributed all possible values that each of the parameters involved in studied problem can take. This has as a result the database to totally reveal the nature of each time studied problem. Furthermore, for experimental databases, especially those composed of data from individual published works, special attention must be paid to ensure that (i) they are published in reputable scientific journals, (ii) they are conducted in certified research laboratories, and (iii) they adhere to all applicable international standards and protocols. Detailed and in-depth works on the principles should be followed during the compilation of a database can be found in^38–41^.

Finally, the database, in addition to being reliable and adequate, should be appended as supplementary materials to every accepted publication. Without the database used for training a computational model, it becomes impossible to justify the reliability of the presented findings. Moreover, it does not promote research, as it forces numerous researchers to compose the entire database without access to previous studies that have been conducted.

#### Glasses preparation

Having the above presented in mind, in this paper, a set of twelve glass series was systematically fabricated by the rapid quenching method. The compositions of each glass batch, meticulously detailed below, were formulated using analytical-grade materials with purities exceeding 99.9% for P_2_O_5_, MgO, CaO, ZnSO_4_, Er_2_O_3_, Sm_2_O_3_, Dy_2_O_3_, TiO_2_, and Ag chemicals. The information concerning the glass sample compositions and codes that relate to them is provided:

- Series PMZxSm: (60 − x)P_2_O_5_ − 20MgO − 20ZnSO_4_ − xSm_2_O_3_, x = 0.5, 1, 1.5, and 2mol%
- Series PMZxDy: (60 − x)P_2_O_5_ − 20MgO − 20ZnSO_4_ − xDy_2_O_3_, x = 0.5, 1, 1.5, and 2mol%
- Series PMZxEr: (60 − x)P_2_O_5_ − 20MgO − 20ZnSO_4_ − xEr_2_O_3_, x = 0.5, 1, 1.5, and 2mol%
- Series PMZSxAg: (59.5 − x)P_2_O_5_ − 20.0MgO − 20.0ZnSO_4_ − 0.5Sm_2_O_3_ − xAg, x = 0.2, and 0.5mol%
- Series PMZDxAg: (59.5 − x)P_2_O_5_ − 20.0MgO − 20.0ZnSO_4_ − 0.5Dy_2_O_3_ − xAg, x = 0.2, and 0.5mol%
- Series PMZExAg: (59.5 − *x*)P_2_O_5_ − 20.0MgO − 20.0ZnSO_4_ − 0.5Er_2_O_3_ − xAg, x = 0.5, 1.0, and 1.5mol%
- Series PMZSAxTi: (60.0 − x)P_2_O_5_ − 20.0MgO − 20.0ZnSO_4_ − 1.0Sm_2_O_3_ − 0.5Ag − xTiO_2_ with x = 0.1, 0.2, 0.3 and 0.4mol%
- Series PZDxCa: (69.0 − x)P_2_O_5_ − 20ZnSO_4_ − xCaO − 1.0Dy_2_O_3_, x = 10, 20, and 30mol%
- Series PMZExTi: (59 − x)P_2_O_5_ − 20MgO − 20ZnSO_4_ − 1Er_2_O_3_ − xTiO_2_, x = 0.1, 0.2, 0.3, 0.4, 0.5, and 0.6mol%
- Series PMZSExTi: (58.0 − x)P_2_O_5_ − 20.0MgO − 20.0ZnSO_4_ − 1.0Sm_2_O_3_ − 1.0Er_2_O_3_ − xTiO_2_, x = 0.2, 0.4, 0.6, 0.8, and 1.0mol%
- Series PMSxZn: (79.0 − x)P_2_O_5_ − xZnSO_4_ − 20.0MgO − 1.0Sm_2_O_3_, x = 10, 20, and 30mol%
- Series PMZETxAg: (58.6 − x)P_2_O_5_ − 20.0MgO − 20.0ZnSO_4_ − 1.0Er_2_O_3_ − 0.4TiO_2_ − xAg, x = 0.01, 0.02, 0.03, 0.04, and 0.05mol%.

**Figure 2:**
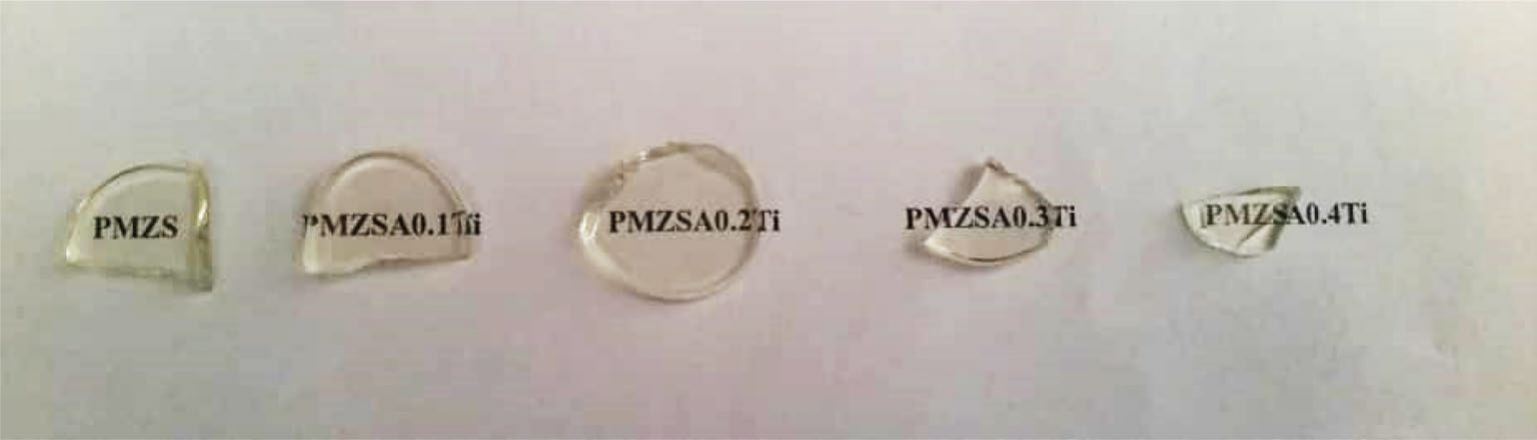
Some of the samples synthesized in this study.

The synthesis process entailed the homogeneous blending of glass constituents, followed by their placement in a platinum crucible. Subsequently, the mixture was subjected to melting within a high-temperature furnace (approximately 1100 °C) for 1 h and 30 min, with periodic stirring. When the molten material reached the appropriate viscosity, it was carefully poured between two stainless steel molds that had been warmed. It was then annealed for 3 h at 300 °C. The as-quenched samples underwent a controlled cooling process within the furnace to room temperature, aimed at minimizing internal stress. The resulting solid specimens frozen were then meticulously polished to acquire optically conducive, precisely flat surfaces. Figure 2 visually confirms the transparency, absence of bubbles, and homogeneity observed in the studied glass samples.

In addition to the laboratory tests, some data were gathered from academic literature^42–44^. Table 1 shows some descriptive statistics chemical elements present in the studied compositions. This study extensively reviews academic literature concerning the empirical determination of three JO parameters (Ω_2_, Ω_4_, Ω_6_) in RE^3+^ doped sulfophosphate glasses. The extracted percentage of oxide compositions, derived from stoichiometry, are compiled for every glass.

**Table 1:** Minimum, median, maximum, and standard deviation of the percentage of oxide compositions obtained over the 60 samples from this study and literature^42–44^.

Element	Min	Max	Mean	Std. deviation
P_2_O_5_	49.00	69.00	58.94	2.76
ZnSO_4_	15.00	20.00	19.65	1.28
MgO	0.00	30.00	19.16	4.36
Nd_2_O_3_	0.00	2.00	0.19	0.51
Ho_2_O_3_	0.00	2.50	0.10	0.43
CaO	0.00	30.00	0.83	4.36
Sm_2_O_3_	0.00	2.00	0.28	0.48
Dy_2_O_3_	0.00	2.00	0.13	0.38
Ag	0.00	1.50	0.10	0.26
Eu_2_O_3_	0.00	2.00	0.09	0.37
Er_2_O_3_	0.00	2.00	0.36	0.51
TiO_2_	0.00	1.00	0.14	0.22
${\Omega }_{2}$	1.97	14.40	7.39	3.30
${\Omega }_{4}$	0.38	12.69	3.53	1.91
${\Omega }_{6}$	0.51	6.90	2.32	1.23

#### Methods

X-ray diffraction (XRD) analyses were conducted utilizing a Bruker D8 Advance diffractometer, employing CuKα radiation (wavelength = 1.54 Å) at 40 kV and 100 mA. The absorption spectra of meticulously polished samples within the range of wavelengths for 250–1640 nm were acquired using a Shimadzu UVPC-3101 spectrophotometer. The data extracted from the UV–Vis absorption edge facilitated the computation of energies for the optical band gap. The refractive index (n) can be expressed with regard to the optical band gap energy (${E}_{opt}$) through the following formula^45^:

9 $$\frac{{n}^{2}-1}{{n}^{2}+2}=1-\sqrt{\frac{{E}_{opt}}{20}}$$

whereas ${E}_{opt}$ represents the optical band gap energy and $n$ denotes the refractive index.

#### Density measurements

Glass density was calculated using the Archimedes method (Precisa Model XT 220 A. Archimedes’), using toluene as the immersion fluid because of its non-hygroscopic and non-reactive properties. The glass density ($\rho$) was defined with the following formula:

10 $$\rho =\frac{{w}_{a}}{{w}_{a}-{w}_{b}}\times {\rho }{\prime}$$

Here, ${w}_{a}$ and ${w}_{b}$ represent the weights of the sample in air and toluene, respectively, and ${\rho }{\prime}$ (0.8669 g cm^−3^) is the density of toluene. The molar volume (${V}_{m}$) of the glass, considering its average molecular weight (${M}_{av}$), is given by:

11 $${V}_{m}=\frac{{M}_{av}}{\rho }$$

where

12 $${M}_{av}=\frac{1}{100}\sum_{i}{x}_{i}{M}_{i}$$

where $x$ and $M$ represent the molar fraction and molar weight of each glass component (*i*).

### Testing procedure

#### XRD pattern

**Figure 3:**
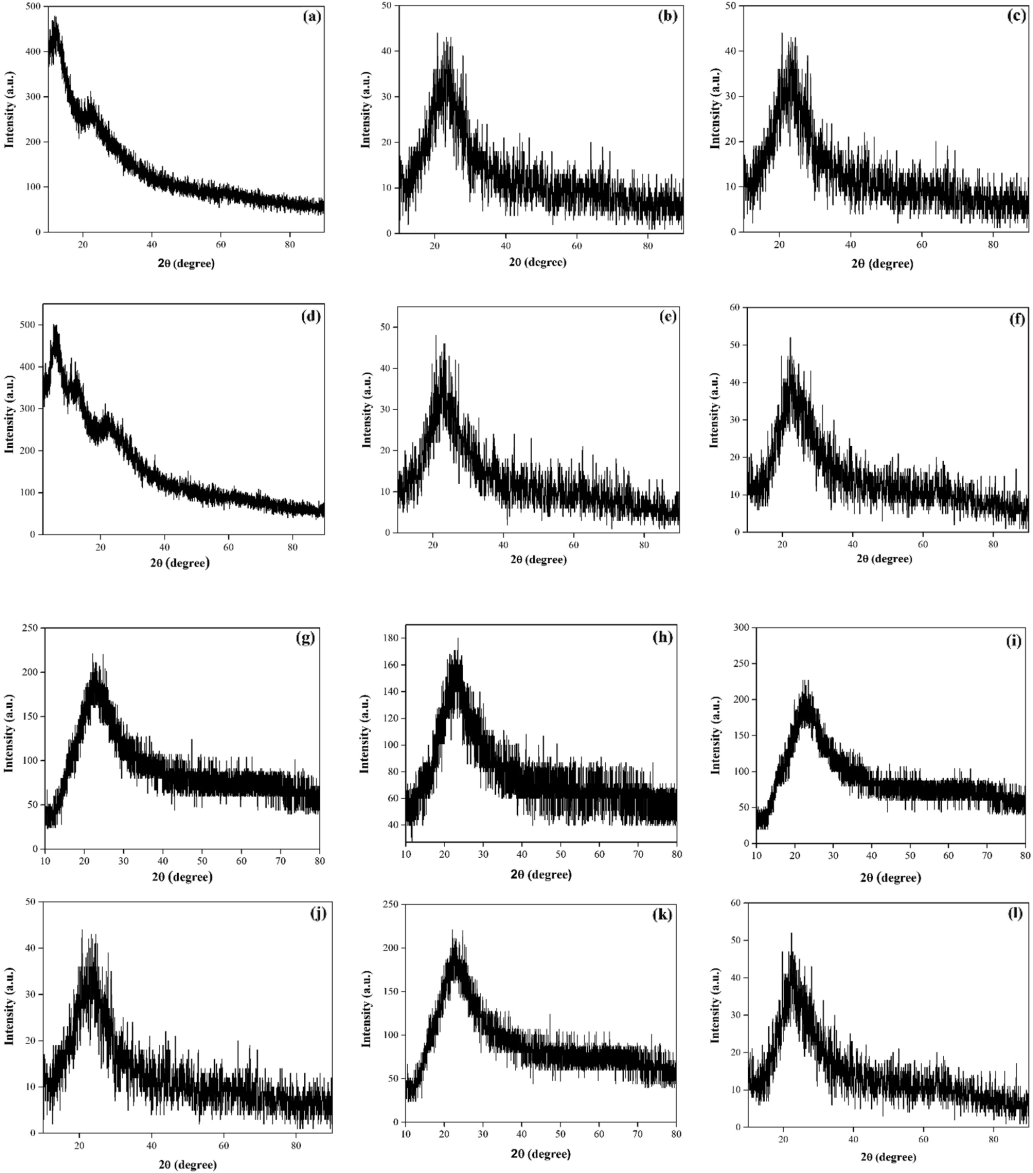
XRD pattern of (**a**) PMZ0.5Sm, (**b**) PMZ0.5Dy, (**c**) PMZ0.5Er, (**d**) PMZS0.5Ag, (**e**) PMZD0.5Ag, (**f**) PMZE0.5Ag, (**g**) PMZSA0.4Ti, (**h**) PZD10Ca, (**i**) PMZE0.5Ti, (**j**) PMZSE0.4Ti, (**k**) PMS30Zn, (**l**) PMZET0.05Ag glass samples.

X-ray diffraction (XRD) analysis was employed to assess the crystalline characteristics of the investigated glasses. Figure 3 illustrates the XRD spectrum for select studied glasses, revealing an absence of discernible diffraction peaks. The long-range structural disorder is indicated by the appearance of a broad peak at a lower scattering angle.

#### Optical properties

**Figure 4:**
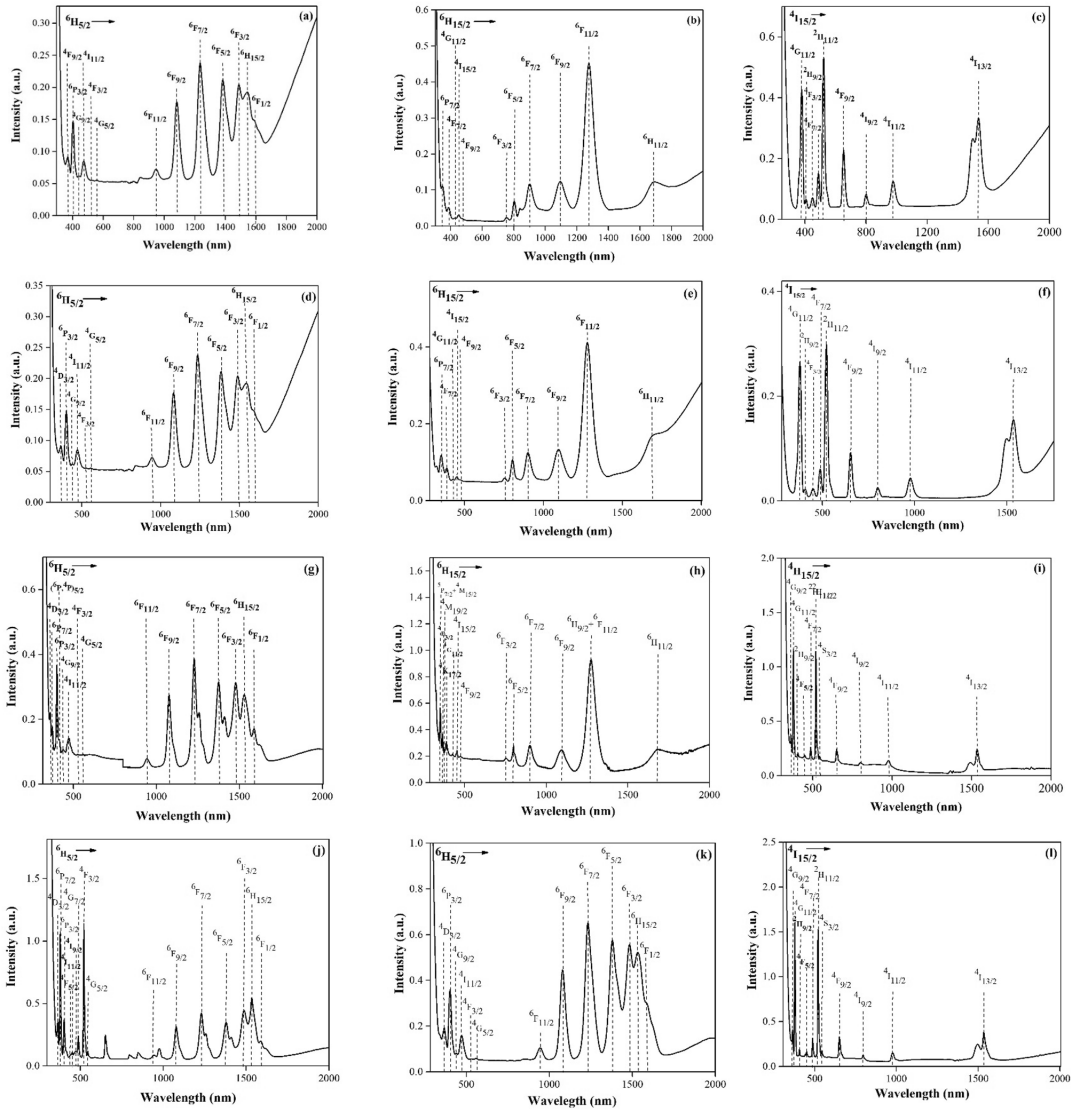
Optical absorption spectrum of (**a**) PMZ0.5Sm, (**b**) PMZ0.5Dy, (**c**) PMZ0.5Er, (**d**) PMZS0.5Ag, (**e**) PMZD0.5Ag, (**f**) PMZE0.5Ag, (**g**) PMZSA0.4Ti, (**h**) PZD10Ca, (**i**) PMZE0.5Ti, (**j**) PMZSE0.4Ti, (**k**) PMS30Zn, (**l**) PMZET0.05Ag glass samples in 300–2000 nm wavelength.

Figure 4 illustrates the absorption spectra of the prepared glass samples within the wavelength range of 300-2000nm. The spectra exhibit distinct absorption bands, each ascribed to rare-earth ions transitions from their ground state to their excited states. These absorption features play a significant part in elucidating the optical characteristics of the glass samples, offering valuable insights into their electronic structure and potential applications in optical devices.

## Development of GEP model

As already mentioned, the key components of a GEP model include the genotype–phenotype mapping, which involves encoding the genetic information into a linear string of symbols and then translating it into a functional program. Developing a GEP model for a problem is a complex iterative process which includes precise definition, selection of appropriate functions and actions, population generation, and development of genetic programs toward optimal solutions. This process requires an understanding of the specific issues of the problem as well as technical knowledge of the GEP algorithm. The developed model should be capable of solving the defined problem with accurate and reliable solutions that requires high precision and expertise. A GEP model can be a powerful tool for solving complicated problems, providing innovative solutions and perspectives that are not possible through traditional methods. A GEP model can also be applied to predict JO parameters which are employed for analysis the spectroscopic properties of rare-earth ions in solid-state materials. With a deep understanding of the JO theory and technical knowledge of GEP, a model can be developed to provide precise and reliable solutions for predicting JO parameters.

To develop the GEP models for predicting JO parameters, 60 datasets were prepared as described in the previous section. To confirm that the results are accurate, existing datasets were randomly separated into two groups of train and test datasets. The train datasets are used for function finding via the GEP models and test datasets, which have no role in training process, are used to evaluate the accuracy of results. Molar percentage (weight % oxide compositions) of all components of the synthesized glasses in the laboratory including 12 components (as shown in Table 1) were chosen to be used as the input data. The JO parameters were also selected to be used as the output parameters. For each output, an optimal model was developed and a mathematical equation was extracted from that model to estimate a JO.

A set of functions and operators that are used to generate genetic programs was selected in order to develop the GEP models. These functions are included mathematical and conditional operators. Then a set of initial population were generated and improved to reach an optimal population. An evaluation of GeneXproTools, a developed code by Ferreira^29^, was employed for the simulations. This code previously was successfully used to simulate engineering problems^46–48^.

The start process of GEP simulation has a random nature which starts with a random creation of the initial population's chromosomes. Consequently, in many cases it does not lead to the desired results and various models with different configurations should be tried to obtain the best possible result. In order to find the best model, following a trial and error approach, numerous models were implemented. In order to choose the best model among the developed models, root mean square error (RMSE) was set as the fitness function. RMSE, as a good tool to obtain prediction errors, is the difference between the value predicted by a model and the actual value. In each model, the fitness between the developed and the measured parameters were compared. In addition, mean absolute error (MAE) and correlation of determination (R^2^) as the conventional statistical criteria of performance measures were obtained for each model. These performance measures can be computed using the following equations:

13 $$RMSE= \sqrt{1/n\sum_{i=1}^{n}{({a}_{i}-{p}_{i})}^{2}}$$

14 $$MAE= 1/n\sum_{i=1}^{n}\left|{a}_{i}-{p}_{i}\right|$$

15 $${R}^{2}={\left(\frac{\sum_{i=1}^{n}\left({p}_{i}-\overline{p }\right)\left({a}_{i}-\overline{a }\right)}{\sqrt{\sum_{i=1}^{n}{\left({p}_{i}-\overline{p }\right)}^{2}\sum_{i=1}^{n}{\left({a}_{i}-\overline{a }\right)}^{2}}}\right)}^{2}$$

Obviously, smaller RMSE and MAE along with higher R^2^ in a model show more reliable estimation. Where, the aim of using these performance measures is to proposed GEP models in lower error with higher correlation. To achieve this goal, following the trial and errors for each JO parameter, several models with different number of train and test dataset as well as different configuration of GEP algorithm were implemented. Tables 2, 3 and 4 show the performance of each implemented model for different JO parameters.

**Table 2:** GEP models implemented for formulation of Ω_2_.

	GEP parameters					Results			
No.	No. of test datasets	Linking function	No. of chromosomes	Head size	Genes' number	RMSE for train datasets	RMSE for test datasets	R^2^ for train datasets	R^2^ for test datasets
1	16	+	12	10	3	1.6537	1.5704	0.7334	0.7510
2	16	×	12	10	3	1.0423	7.2033	0.8943	0.4803
3	17	+	16	12	5	1.2837	2.9813	0.8495	0.5826
4	17	×	16	12	5	0.6100	2.7332	0.9634	0.3943
5	18	+	20	14	6	1.0736	1.6420	0.8987	0.7538
6	18	+	20	14	7	0.9494	2.3725	0.9114	0.6582
7	18	×	20	14	7	1.6790	2.0624	0.7379	0.6308
8	19	+	26	16	4	1.1151	2.2205	0.8835	0.5639
9	19	+	26	16	6	1.2882	1.7167	0.8369	0.7425
10	19	+	32	16	8	1.0141	4.0280	0.9022	0.5386
11	19	×	26	16	6	0.9926	3.2393	0.9234	0.4492
12	19	×	32	16	8	1.3855	1.7350	0.8096	0.7374
13	20	+	36	18	6	0.8760	0.9665	0.9152	0.9078
14	20	+	40	18	6	0.6549	0.6849	0.9618	0.9507
15	20	×	36	18	6	0.8287	1.1516	0.9256	0.9076
16	20	×	40	18	6	1.0576	0.7232	0.9093	0.9478
17	21	+	44	20	8	1.0122	0.8581	0.9009	0.9269
18	21	+	48	22	8	0.7930	0.8737	0.9312	0.9375
19	21	×	48	22	8	0.9149	1.2908	0.9468	0.8372
20	22	+	40	18	6	0.6948	0.7106	0.9479	0.9499
21	22	+	44	20	8	0.8947	1.7590	0.9107	0.7040
22	22	×	44	20	8	1.0840	1.6828	0.8133	0.6727
23	23	+	40	18	6	1.1041	1.8122	0.8633	0.6846
24	23	×	40	18	6	1.4955	1.9729	0.7415	0.6232
25	24	+	40	18	6	1.9677	2.1566	0.6434	0.5638

**Table 3:** GEP models implemented for formulation of Ω_4_.

	GEP parameters					Results			
No.	No. of test datasets	Linking function	No. of chromosomes	Head size	Genes' number	RMSE for train datasets	RMSE for test datasets	R^2^ for train datasets	R^2^ for test datasets
1	19	+	25	12	3	0.7013	0.5976	0.8368	0.7524
2	19	+	30	12	3	0.4235	0.5940	0.9422	0.7829
3	19	×	30	12	3	0.4214	0.6268	0.9409	0.7384
4	20	+	35	13	5	0.3754	0.4733	0.9551	0.8799
5	20	×	35	13	5	0.4765	0.4854	0.9311	0.7762
6	20	+	40	18	6	0.4127	0.3867	0.9400	0.9106
7	20	×	40	18	6	0.4424	0.3332	0.9410	0.8974
8	21	+	40	15	8	0.2987	0.3149	0.9722	0.9274
9	21	×	40	18	8	0.4016	0.7037	0.9029	0.8937
10	21	+	40	16	8	0.2594	0.4838	0.9466	0.8854
11	21	×	40	16	8	0.3291	0.5355	0.9363	0.9121
12	22	+	41	15	8	0.3785	0.5857	0.9098	0.7240
13	22	+	42	15	8	0.3390	0.5075	0.9380	0.8097
14	22	+	43	15	9	0.3328	0.5275	0.9324	0.7886
15	22	×	43	15	9	0.3780	0.4736	0.9028	0.8875
16	23	+	44	15	8	0.3687	0.4194	0.8760	0.8888
17	23	×	44	15	8	0.3081	0.5025	0.9007	0.8217
18	23	+	45	16	9	0.4271	0.3242	0.8462	0.8869
19	23	×	45	16	9	0.3756	0.4332	0.8958	0.8129
20	24	+	40	18	8	0.4144	0.3766	0.8710	0.8160
21	24	×	40	18	8	0.3288	0.5072	0.9079	0.7888
22	24	+	40	20	10	0.7568	1.0106	0.6791	0.7755
23	24	×	40	20	10	0.3622	0.5174	0.8937	0.8546
24	25	+	40	22	10	0.3697	0.5136	0.9172	0.7028
25	25	×	40	22	10	0.3509	0.3115	0.9114	0.8837

**Table 4:** GEP models implemented for formulation of Ω_6_.

	GEP parameters					Results			
No.	No. of test datasets	Linking function	No. of chromosomes	Head size	Genes' number	RMSE for train datasets	RMSE for test datasets	R^2^ for train datasets	R^2^ for test datasets
1	17	+	12	10	6	0.7214	0.7639	0.7316	0.6161
2	17	×	12	10	6	0.7441	0.7421	0.7648	0.6434
3	18	+	15	12	8	0.6841	0.6517	0.7666	0.7074
4	18	×	15	12	8	0.5303	0.4811	0.8629	0.7512
5	19	+	20	14	8	0.8995	0.7311	0.6500	0.7092
6	19	×	20	14	8	0.7740	0.6840	0.7486	0.6618
7	20	+	20	18	6	0.5048	0.9539	0.8707	0.8019
8	20	+	30	14	8	0.5362	0.7348	0.7746	0.6956
9	20	×	40	18	6	0.4899	0.4570	0.8402	0.8592
10	21	+	35	14	6	0.4706	0.4207	0.8241	0.8270
11	21	+	40	15	8	0.4815	0.8040	0.8308	0.8258
12	22	+	40	16	8	0.4936	0.4143	0.8684	0.8605
13	22	×	40	16	8	0.4466	0.5530	0.8903	0.8600
14	23	+	45	18	6	0.4323	0.4032	0.8915	0.8672
15	23	+	45	20	8	0.4259	0.5834	0.8375	0.8335
16	24	+	45	22	10	0.3558	0.3287	0.9174	0.8979
17	25	×	45	22	12	0.3158	0.5763	0.9208	0.9158
18	19	+	30	10	12	0.2356	0.2042	0.9686	0.9473
19	19	+	35	10	12	0.2124	0.3387	0.9618	0.9080
20	19	×	40	10	12	0.4145	0.3464	0.8674	0.9139
21	20	+	40	12	12	0.3221	0.6439	0.9097	0.9119
22	20	+	45	10	12	0.4234	0.3975	0.8918	0.9079
23	20	×	45	12	12	0.4262	0.4905	0.8786	0.8983
24	21	+	30	12	14	0.6265	0.7527	0.8515	0.8249
25	21	+	30	14	14	0.7688	0.3642	0.8756	0.8844

Selection the models were conducted based on the evaluation of performance measures. Eventually, models No. 14 from Table 2, No. 8 from Table 3 and No. 18 from Table 4 were selected as the optimal models. The configuration settings of the GEP algorithm for the selected models are tabulated in Table 5.

**Table 5:** Configuration settings for GEP algorithm.

	Parameter	Ω_2_	Ω_4_	Ω_6_
General	No. of chromosomes	40	40	30
	head size	18	15	10
	No. of genes	6	8	12
	linking function	Addition	Addition	Addition
	function set	+ , −, *, /, Exp, Ln, Inv, x^2^, 3Rt, Min 2, Ave 2, Max 2, Atan, Tanh, NOT	+ , −, *, /, Pow, Sqrt, Exp, Pow10, Ln, Log, Inv, x^2^, x^3^, x^4^, x^5^, 3Rt, 4Rt, 5Rt, Min 2, Ave 2, Max 2, Atan, Tanh, NOT	+ , −, *, /, Pow, Sqrt, Exp, Pow10, Ln, Log, Abs, Inv, x^2^, x^3^, x^4^, 3Rt, 4Rt, Min 2, Max, 2Ave 2, Ave 3
	Fitness function	RMSE	RMSE	RMSE
Genetic operators	Mutation rate	0.00138	0.00138	0.00138
	IS transposition rate	0.00546	0.00546	0.00546
	RIS transposition rate	0.00546	0.00546	0.00546
	Inversion rate	0.00546	0.00546	0.00546
	Gene recombination	0.00277	0.00277	0.00277
	Gene transposition	0.00277	0.00277	0.00277
Numerical constants	Constants per gene	10	10	10
	Data type	Floating-point	Floating-point	Floating-point
	Lower bound	− 10	−10	−10
	Upper bound	+ 10	+ 10	+ 10

## Results and discussions

As mentioned in the previous section, three models were developed separately to predict JO parameters. The setting, performance and results of each model for both train and test datasets are presented in Tables 2, 3 and 4 for the variables of Ω_2_, Ω_4_ and Ω_6_, respectively. In all steps, the learning ability of the models was specified in the training process while the performance of each model that shows its ability to be used in practice was evaluated in the testing procedure. Accordingly, model performance evaluation criteria including R^2^ and RMSE were used to estimate the performance of each model to find the more accurate model.

To develop a model with an acceptable performance the GEP configuration settings, as influential parameters, were also changed in each model. From the tables, it can be observed that a change in the GEP parameters does not create the same increasing or decreasing trend in the model performance which is due to the nature of the GEP modeling process. Due to the large number of input variables considered for JO parameters prediction, finding a high-performance model is very complicated. Hence, the models with various head size were evaluated as the head size determines the complication of each variable in a developed model.

**Figure 5:**
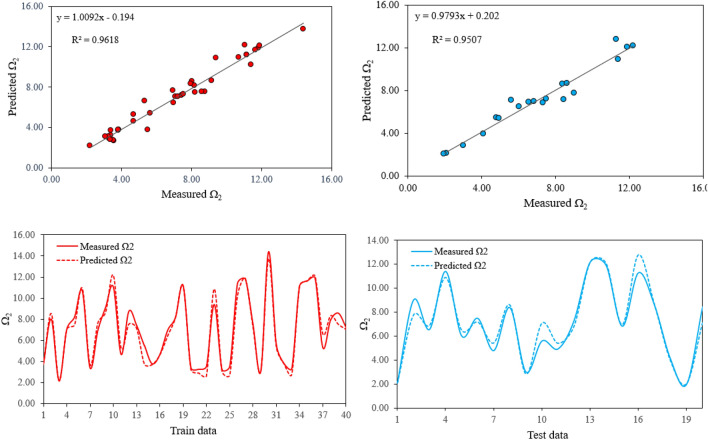
Comparison between predicted Ω_2_ values by the proposed model and the experimental measured Ω_2_ for train (right) and test (left) datasets.

To predict Ω_2_, model No. 8 was chosen as the best model among all models of Table 2. In this model, 40 train and 20 test datasets, 40 chromosomes with 18 head size and 6 genes were employed. With an R^2^ of 0.96 for train and 0.95 for test datasets, this model has performed better among all models. It should be mentioned that R^2^ value alone is not enough to evaluate the accuracy of a model. Therefore, RMSE as the error evaluation index was also used. RMSE values 0.6549 and 0.6849 were respectively obtained for train and test datasets of the selected model. Figure 5 shows an illustrative comparison between predicted Ω_2_ values by the proposed model and the experimental Ω_2_ measures for train and test datasets. According to these figures, predicted Ω_2_ values are in good agreement with the experimental Ω_2_ measured values which indicate the capability of the proposed model to predict JO parameters.

An equation can also be extracted from this model to be used for prediction of Ω_2_. This equation can be presented in the form of a mathematical equation or a computer program code. Considering that 14 variables were used as the input parameters, a complex equation is derived to predict the value of Ω_2_ from the selected model. Therefore, this equation is presented in the form of a Matlab code, which makes its use very simple. This code can be found in Appendix A1.

**Figure 6:**
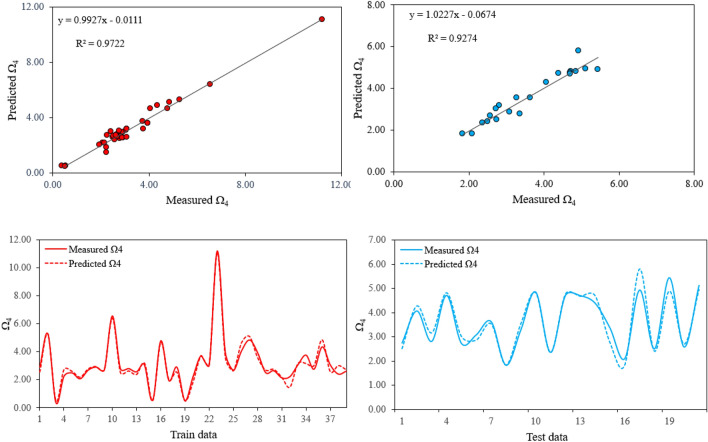
Comparison between predicted Ω_4_ values by the proposed model and the experimental measured Ω_4_ for train (right) and test (left) datasets.

**Figure 7:**
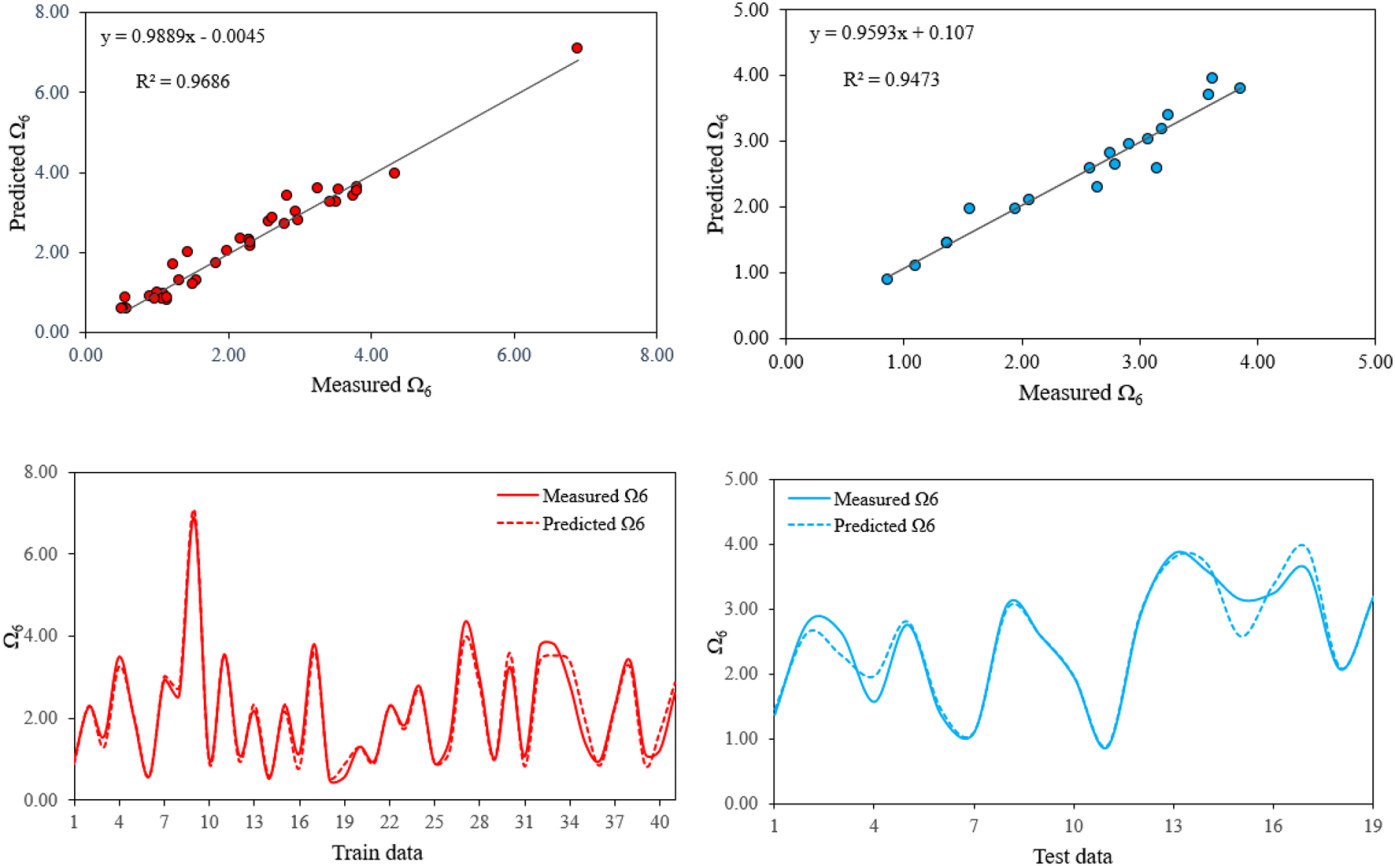
Comparison between predicted Ω_6_ values by the proposed model and the experimental measured Ω_6_ for train (right) and test (left) datasets.

Similarly, models No. 8 and No. 18 were respectively selected to predict Ω_4_ and Ω_6_ from Tables 3 and 4. Figures 6 presents the experimental vs predicted values for the Ω4 parameter while Fig. 7 presents the experimental vs predicted values for the Ω6 parameter. The strong correlation between predicted and measured values of Ω_4_ and Ω_6_ (i.e. R^2^ = 0.97 for train and R^2^ = 0.93 for test datasets of Ω_4_ and R^2^ = 0.97 for train and R^2^ = 0.95 for test datasets of Ω_6_) besides the acceptable errors (i.e. RMSE = 0.2987 for train and RMSE = 0.3149 for test datasets of Ω_4_ and RMSE = 0.2356 for train and RMSE = 0.2042 for test datasets of Ω_6_) indicates the good generalization performance of the proposed models. Two Matlab codes were also extracted from the proposed models which can be easily used to predict JO Ω_4_ and Ω_6_ parameters. These codes can be seen in Appendix A2 and A3. The possibility of using the extracted codes from the proposed GEP models which can be quickly and easily done with acceptable accuracy, makes them a useful tool for predicting JO parameters. However, it is deserved to applied the proposed models as preliminary estimates and cautiously be used for the final stages.

## Limitations and future perspectives

In this section, the limitations and immediate research prospects of the authors are presented based on the results of the present study, which have been discussed above, and the main conclusions will be presented below. It is worth writing that any forecasting soft computing mathematical model is valid for parameter values (input parameters) that fall within the minimum and maximum values that each parameter takes based on the experimental database used for its design, development, and training. Thus, the proposed Gene Expression Programming optimal models, which have been developed and presented herein, are valid for values between the minimum and maximum for each input parameter as presented in Table 1.

A primary priority among the authors' future research objectives is to update the database with a larger number of datasets covering statistically uniformly all possible values of the input parameters, thus making the estimation of the optical properties of rare-earth doped phosphate glasses more reliable revealing their complicated and strongly nonlinear nature.

## Conclusion

The JO theory stands as a pivotal framework in rare-earth spectroscopy, holding implications across various scientific disciplines. Its role in the spectroscopic characterization of materials places it at the core of material science and chemistry. However, accurate estimation of the three principal parameters, Ω_2_, Ω_4_, and Ω_6_, necessitates extensive experimental work. Moreover, the mathematical intricacies involved render such inferences challenging for non-experts and particularly inaccessible for experimentalists with limited knowledge of quantum mechanics. These obstacles to optical material innovation serve as a deterrent. Statistical and chemometric methods were used in an attempt to determine the illusive parameters without the usual difficult experimental and theoretical processes. The objective was to establish a relationship between accessible information regarding the materials of interest and the related JO parameters, generating subsequent optical characterizations. Remarkably, by solely considering the bulk composition of a limited number of sulfophosphate glasses doped with RE^3+^ (from literature and experimental work), it successfully estimated the parameters with a proper margin of error. This estimation, previously only possible through complicated experimental and analytical procedures, represents a significant achievement. Interestingly, predicted Ω_2_ values are consistent with experimental findings of Ω_2_ values, indicating the proposed model can accurately predict JO parameters. The strong correlation between predicted and measured values of Ω_4_ and Ω_6_ (i.e. R^2^ = 0.97 for train and R^2^ = 0.93 for test datasets of Ω_4_ and R^2^ = 0.97 for train and R^2^ = 0.95 for test datasets of Ω_6_) besides the acceptable errors (i.e. RMSE = 0.2987 for train and RMSE = 0.3149 for test datasets of Ω_4_ and RMSE = 0.2356 for train and RMSE = 0.2042 for test datasets of Ω_6_) specifies the good generalization performance of this models.

In conclusion, this study not only addresses a pressing challenge in materials science but also demonstrates the transformative potential of advanced computational techniques like GEP. By bridging the gap between theory and experiment, this research paves the way for accelerated innovation in the field of optical materials, showcasing the collaborative efforts of researchers from diverse scientific backgrounds and serving as a valuable educational resource.

## Data Availability

Data will be made available upon request from the corresponding author.
